# ﻿Identification and distribution of leafrollers (Lepidoptera, Tortricidae) associated with berries (Rosaceae) cultivated in Mexico

**DOI:** 10.3897/zookeys.1146.81734

**Published:** 2023-02-09

**Authors:** Isabel Ruiz-Galván, Néstor Bautista-Martínez, Lauro Soto-Rojas, Samuel Pineda-Guillermo, Jesús Romero-Nápoles

**Affiliations:** 1 Colegio de Postgraduados Campus Montecillo, Instituto de Entomología y Acarología, km. 36.5 Carretera México-Texcoco, Montecillo, C.P. 56230, Texcoco, Estado de México, Mexico Instituto de Entomología y Acarología Texcoco Mexico; 2 Instituto de Investigaciones Agropecuarias y Forestales, Universidad Michoacana de San Nicolás de Hidalgo, Morelia, Michoacán, Mexico Universidad Michoacana de San Nicolás de Hidalgo Morelia Mexico

**Keywords:** Altitude, blackberry, damage, genitalia, raspberry, strawberry, tortricids

## Abstract

Berries are agricultural products of great economic interest for Mexico, and their production has increased in recent years; however, crops are affected by tortricid leafrollers. From August 2019 to April 2021 in Michoacán and Guanajuato, Mexico, a study was conducted to determine the species of tortricids associated with blackberries (*Rubus* spp. L.), raspberries (*Rubusidaeus* L.) and strawberries (*Fragaria×ananassa* Duch.), as well as their altitudinal distribution. In 12 orchards located in these states, shoots, leaves and flowers infested by larvae were collected. The species were identified by male genitalia and were determined taxonomically as *Amorbiacuneana* (Walsingham, 1879), *Argyrotaeniamontezumae* (Walsingham, 1914) and *Platynota* sp. Walker, 1859, found at elevations from 1290 to 2372 m. The most abundant species were *A.cuneana* and *A.montezumae*. Generally, these tortricids prefer to feed on tender vegetative parts of the plant, but the economic impact they have is not known. It is worth mentioning that the number of species found is lower than those reported in other countries, but it is necessary to broaden the study area to other berry-producing regions to determine whether their distribution is wider.

## ﻿Introduction

The small fruits (berries) of the family Rosaceae include blackberries (*Rubus* spp. L.), raspberries (*Rubusidaeus* L.) and strawberries (*Fragaria×ananassa* Duch.). The family is widely distributed although is better adapted to temperate climates (Rzedowski 2021). The Mexican states where production of these berries is concentrated are mainly Michoacán, Jalisco, Baja California, and Guanajuato (SIAP 2021). According to data from FAO-FAOSTAT (2021), Mexico is situated among the first five berry-producing countries of the world, and production has increased in the last 15 years. In 2020, Mexico exported more than US$1989 million in berries (SIAVI 2021).

As in other crops, this group of berries is affected by pests that limit production. The family Tortricidae (microlepidoptera) is one of the most diverse of Lepidotera. It is divided into three subfamilies, Tortricinae, Olethreutinae, and Chlidanotinae (Gilligan and Epstein 2014), that together include approximately 11,500 species and 1787 genera (Gilligan et al. 2018; Gilligan et al. 2020). The number of tortricid agricultural pests worldwide is estimated at 700 species (Gilligan and Epstein 2014), although there are undescribed species. The distribution of the family is cosmopolitan, although it is better adapted to temperate, subtropical, and tropical climates (Meijerman and Ulenberg 2000). In general, species of Tortricinaehave a polyphagous habit, while most Olethreutinae are oligophagous. They feed on approximately 12,000 species, including vegetable, fruit, ornamental and forest crops (Hill 1987; Brown et al. 2008). Tortricids, are commonly known as leaf rollers because the larvae feed often on foliage, produce silk, and shelter in rolled leaves while they feed. They have also been found defoliating or boring into shoots, flowers and fruits of diverse plant species (Brown et al. 2008).

Some species of microlepidoptera are of major economic importance and may cause total production loss (Akbarzadeh 2012). Gilligan et al. (2020) argue that, of the total number of Lepidoptera introduced into North America, 23% to 30% are tortricids. The compilation by Brown et al. (2008) presents 97 species of tortricids associated with *Rubus* spp. and 52 species associated with *Fragaria* spp. worldwide. Among reported leaf roller hosts are species of Rosaceae, such as the genera *Rubus* and *Fragaria* sp. (McQuillan 1992; Brown et al. 2014, 2019) with records of their association in regions of Australia, Asia, Europe and North America (Brown et al. 2008).

Knowledge of diversity is fundamental in fauna research (Luis-Martínez et al. 2020), including determination of a species geographic distribution, its association with its hosts, and its ecological biogeography (Arita and Rodríguez 2001). Monteagudo-Sabaté et al. (2001) consider altitude to be one of the most important components in species determination. Sanders (2002) stated that greater species diversity occurs at low altitudes. In contrast, the studies of McCoy (1990) suggest that greater richness occurs at middle altitudes.

Despite the diversity of tortricids reported in berries in other regions of the world and the economic importance of berries, knowledge of the interaction of this group of insects and plants is scarce. Only López et al. (2014), Martínez et al. (2014), and Juárez-Gutiérrez et al. (2015) have reported *Argyrotaeniamontezumae* (Walsingham, 1914) and *Amorbiacuneana* (Walsingham, 1879) in blackberries (*Rubusidaeusalis*), while Tejeda-Reyes et al. (2020) reported *A.montezumae* in strawberries (*Fragaria×ananassa*). Worldwide, ecosystems are transforming at an accelerated pace, and for this reason, determining species in unexplored areas is a priority.

Therefore, the objective of this study was to identify the species of tortricids that feed on berries of Rosaceae along an altitudinal gradient from 1290 to 2337 m in Michoacán and Guanajuato, Mexico.

## ﻿Material and methods

### ﻿Sampling sites and collection of plant material

The study was conducted from August 2019 to April 2021 in Michoacán and Guanajuato, Mexico (Table 1). The commercial crops sampled were (*Rubus* spp.) varieties ‘Tupy’ and ‘brazos’, raspberry (*Rubusidaeus* L.) variety ‘Meerker’, and strawberry (*Fragaria×ananassa* Duch.) variety ‘Camino real’. The orchards were located at elevations from 1290 to 2337 m, with average annual temperature of 21 °C, and a warm temperate climate (García 1998). In each orchard, 1 ha of the crop was sampled in linear rows. Shoots and leaves with evidence of leafroller larvae were collected. Three to 12 plant parts were collected on each sampling date, depending on the abundance of larvae. The phenological phases of the crops were vegetative development, flowering, and fruit set.

**Table 1.** Tortricids identified in blackberry, raspberry, and strawberry orchards in Guanajuato and Michoacán, Mexico. Number of emerged adults in parentheses.

State	Municipality	Crop	Altitude (m)	Coordinates	Species	Plant part attacked	Sampling date
Michoacán	Los Reyes	Blackberry	1290	19.5944, -102.4885	*Platynota* sp. (1♂)	Leaf bud	9-IX-2019
							02-X-2019
					*Amorbiacuneana* (6♂, 5♀)	Leaf bud	15-X-2019
	Peribán		1372	19.5510, -102.4609	*Amorbiacuneana* (3♂)	Leaf bud	02-X-2019
							16-X-2019
					*Argyrotaeniamontezumae* (1♂)	Leaves	20-XI-2019
	Tangancícuaro		1702	19.8986, -102.1939	*Amorbiacuneana* (2♂)	Leaf bud	02-IX-2019
							01-X-2019
					*Argyrotaeniamontezumae* (2♂)	Leaves	15-X-2019
							18-XI-2019
			1739	19.8589, -102.2109	*Amorbiacuneana* (3♂, 3♀)	Leaf bud	09-IX-2019
							01-X-2019
					*Argyrotaeniamontezumae* (2♂)	Leaves	15-X-2019
							18-XI-2019
		Raspberry	1707	19.8903, -102.1794	*Argyrotaeniamontezumae* (2♂)	Leaf bud	15-X-2019
						Leaves	18-XI-2019
	Maravatío	Blackberry	2030	19.8911, -100.3578	------*	Leaf bud	18-X-2019
						Leaves	22-XI-2019
							04-XII-2019
			2031	19.8920, -100.3564	*Amorbiacuneana* (1♂, 1♀)	Leaf bud	18-X-2019
							22-XI-2019
					*Argyrotaeniamontezumae* (1♀, 3♂)	Leaves	04-XII-2019
	Villa Madero	Raspberry	1650	19.4160, -101.2307	*Argyrotaeniamontezumae* (1♂)	Leaves	17-X-2019
							22-XI-2019
							02-XII-2019
			2337	19.3832, -101.3235	*Amorbiacuneana* (2♀, 2♂)	Leaf bud	17-X-2019
							22-XI-2019
					*Argyrotaeniamontezumae* (3♀, 2♂)	Leaves	02-XII-2019
Guanajuato	Jaral del Progreso	Raspberry	1723	20.4199, -101.0595	*Amorbiacuneana* (2♀)	Leaf bud	30 IX-2020
					*Argyrotaeniamontezumae* (2♀)		14-IV-2021
	Victoria de Cortázar		1729	20.3421, -101.0287	*Amorbiacuneana* (2♀, 3♂)	Leaf bud	30-IX-2020
					*Argyrotaeniamontezumae* (2♀)		14-IV-2021
	Jaral del Progreso	Strawberry	1724	20.3756, -101.0501	*Amorbiacuneana* (1♀)	Leaves	30-IX-2020
					*Argyrotaeniamontezumae* (2♀, 1♂)		14-IV-2021

*Collected larvae that did not complete development to adult stage.

Infested plant organs were cut into lengths of 10 to 15 cm. Each plant part was conditioned individually in a Num. 4 plastic cup (Reyma, Mexico) with water and sponge. A “plastic cage” constructed with two 1-L plastic cups joined at the edges was later introduced. The upper cup had organza fabric (Parisina, Mexico) on the bottom. Each sample was labeled with collection data. The collected material was transported to the Entomology Laboratory of the Colegio de Postgraduados, Campus Montecillo, Texcoco, State of Mexico, where they were kept at a temperature of 25±2 °C, 60 ± 20% relative humidity and photoperiod of 12:12 h (light/dark) until adult emergence.

### ﻿Species identification

Adults were separated by sex and morphotypes, mounted and labeled. The specimens were identified by comparing male genitalia, with illustrations, literature, and taxonomic keys of Obraztsov (1961), Mackay (1962), Phillips-Rodríguez and Powell (2007), Razowski et al. (2008), Trematerra and Brown (2004), Brown (2013), Gilligan and Epstein (2014) and Gilligan et al. (2018). In addition, identification was corroborated by taxonomists specialized in Tortricidae, Dr John W. Brown (National Museum of Natural History, Washington D.C. USA) and Dr Jason Dombroskie (Insect collection of Cornell University, Ithaca, NY, USA). The genitalia were photographed with a Photomicroscope III Carl Zeiss (Carl Zeiss, Germany). Larva and adult specimens of the species found are located in the Entomological Collection of the Institute of Plant Health (CEAM), Colegio de Postgraduados, Campus Montecillo, Texcoco, State of Mexico, Mexico.

## ﻿Results

We collected 255 plant parts with larvae; of these 85% were blackberry, 10% raspberry and 5% strawberry. We identified three species of tortricids: *Argyrotaeniamontezumae* (Tortricinae: Archipini), and *Amorbiacuneana* and *Platynota* sp. (Tortricinae: Sparganothini). *Amorbiacuneana* was the most abundant species in the three crops, accounting for more than 60% of all the species found during the study period. The different species were distributed over all the altitudes studied, from 1290 to 2337 m. Nevertheless, we observed that *A.montezumae* preferred higher altitudes. Table 1 presents the number of emerged adults at each site and their host.

### ﻿Damage

**Figure 1.**
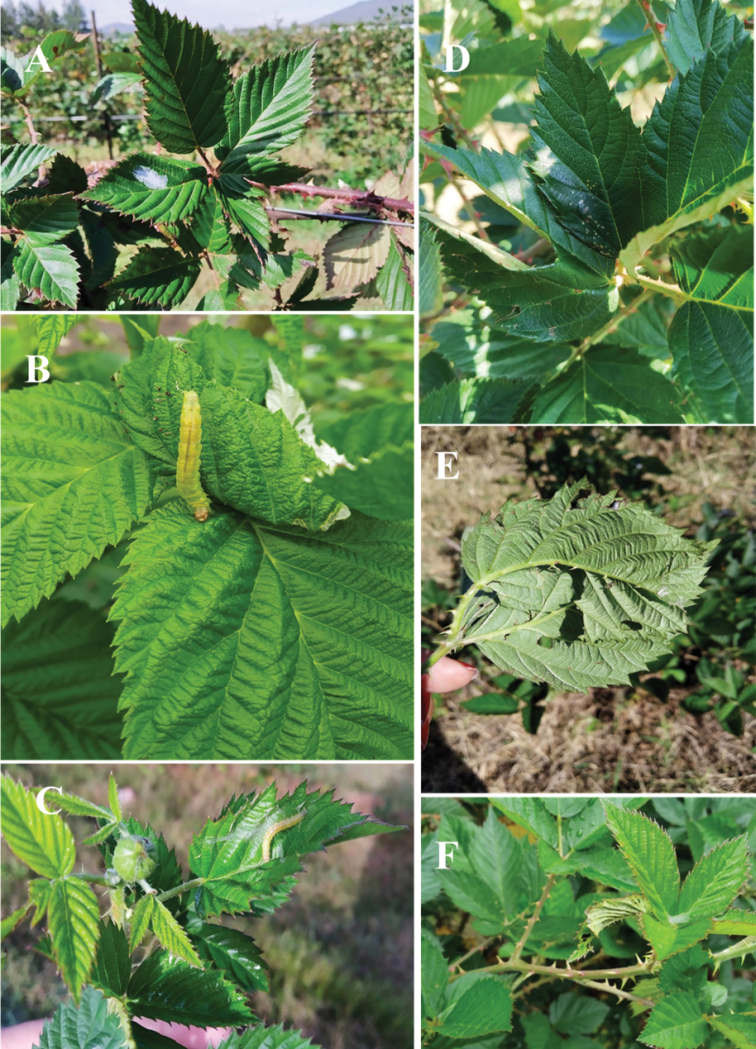
Damage caused by leafrollers in berries **A***A.cuneana* egg mass in blackberry **B***A.montezumae* in a raspberry shoot **C** silk produced by a larva on a leaf **D** and **E** folded leaves with a larva inside **F** leaf rolled toward the face.

The leafrollers *A.cuneana* and *A.montezumae* oviposit in flattened oval masses of more than 100 eggs on the face of the leaves and near the central vein. *Amorbiacuneana* covers the egg mass with a white secretion that extends beyond the mass (Fig. 1A), while *A.montezumae* oviposits the eggs superimposed and uncovered. When the eggs hatch, the larvae disperse, actively searching for a feeing site. Cannibalism among *A.cuneana* larvae is evident since they are found isolated on the same plant in the crop and it was observed in the field and laboratory (Espino-Herrera et al. 2012).

Larvae of both species feed on tender developing leaves (Fig. 1B). They join the lateral edges of the leaves with silk (Fig. 1C) and form a shelter of joined leaves where small perforations can be observed (Fig. 1D, E) or a leaf rolled into a “turnover” shape (Fig. 1F). Only one larva is found in each shelter where it feeds, protects itself and pupates.

## ﻿Discussion

Brown et al. (2008) presents 97 species of tortricids that are associated with the genus *Rubus* spp. and 52 species with *Fragaria* spp. worldwide. Therefore, the three species found in blackberry, raspberry and strawberry at altitudes between 1290 and 2337 m constitute only 2% of the species richness of Tortricidae in these hosts in Mexico. These three species are only a small fraction of the 25 and 24 species of microlepidoptera reported on the American continent associated with *Rubus* and *Fragaria*, respectively (Hill 1987; Brown et al. 2008).

Records of *Amorbia* spp., *Argyrotaenia* spp. and *Platynota* spp. in crops are scarce in Mexico. Juárez-Gutiérrez et al. (2015) registered the presence of *A.cuneana* in blackberry (*Rubus* sp.) in Michoacán, while Urías-López and Salazar-García (2008) registered this same species in avocado (*Perseaamericana* Miller) in Nayarit. Rosas and Villegas (2008) reported that *Argyrotaenia* sp. feeds on avocado foliage and fruits in Nayarit and Michoacán. *Argyrotaeniamontezumae* has also been reported in blackberries (López et al. 2014; Martínez et al. 2014; Barreto et al. 2016), in strawberries (Tejeda-Reyes et al. 2020), and hawthorn (*Crataegusmexicana* Moc. & Sessé ex DC.) (Tejeda-Reyes et al. 2021). Varela-Fuentes et al. (2009) identified *Platynotarostrana* Walker (1863) feeding on Valencia orange (*Citrussinensis* L. Osbeck) and lemon (*Citruslimon* (Linnaeus) N.L. Burman) in Tamaulipas, while Bautista et al. (2014) argue that *Platynota* sp. feeds on *Opuntia* spp. in the state of Mexico.

**Figure 2.**
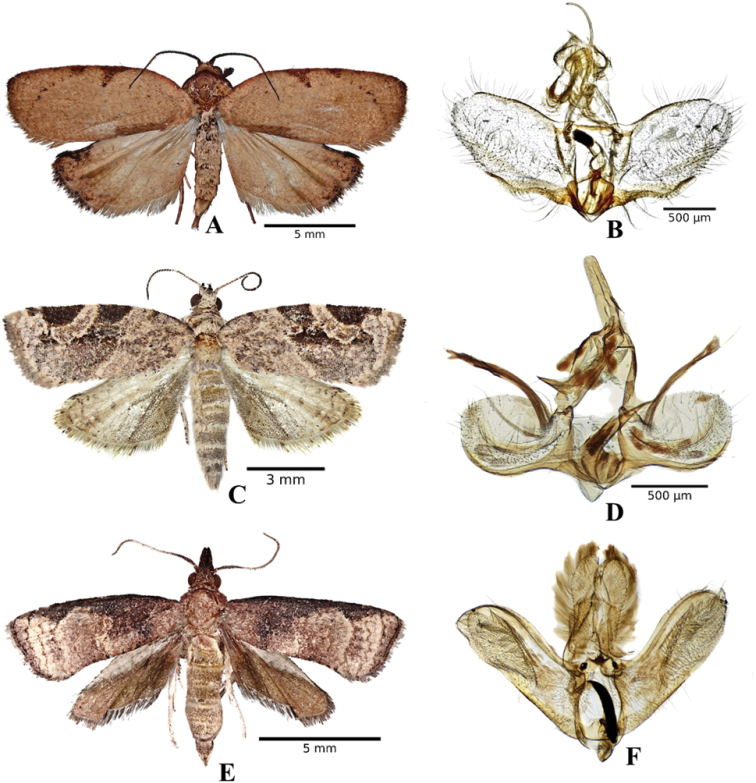
**A** and **B***Amorbiacuneana*, Tangancícuaro, Michoacán **C** and **D***Argyrotaeniamontezumae*, Peribán, Michoacán **E** and **F***Platynota* sp., Los Reyes, Michoacán.

Adult *Amorbia* are one of the largest tortricid moths in North America. They are generally distinguished by a diffuse pattern on their forewings (Powell and Brown 2012) and by the fenestra on the dorsal abdominal segments (Phillips-Rodríguez and Powell 2007): in segments 2 to 6 for *A.emigratella* and only one in segment 2 for *A.cuneana* (Gilligan and Epstein 2014). However, it is essential to look at more specific structures for their identification. The masculine genitalia of *A.cuneana* and *A.emigratella* are similar, but traits such as the less pronounced basal expansion of the uncus in *A.emigratella* and the slightly narrower distal half of the valva, and the different articulation of the base of the uncus with the dorsal of the tegumen in *A.cuneana* (Fig. 2B), are highly useful for separating these two species (Powell and Brown 2012).

In our study, *A.cuneana* was found feeding on raspberry and blackberry leaf buds and on strawberry leaves in 10 of the 12 sampled orchards at elevations of 1290 to 2337 m, coinciding with Juárez-Gutiérrez et al. (2015) who reported *A.cuneana* feeding on blackberry leaves and with Powell and Brown (2012) who report this genus at altitudes of 2500 m in California. The highest species richness of the *Amorbia* species is reported at elevations of 500–1500 m (Phillips-Rodríguez and Powell 2007). Gilligan and Epstein (2014) highlight that this tortricid has been registered feeding on 34 genera of plants belonging to 25 families, including *Rubus* spp. as economically important crops. Moreover, the compilation of Brown et al. (2008) reveals that *Fragaria* spp. has not been registered as a host to any species of *Amorbia*. This has been ratified by Powell and Brown (2012) and Gilligan and Epstein (2014). Therefore, this is the first report of association between strawberry (*F.×ananassa*) and *A.cuneana*, whose larvae were found rolling young strawberry leaves in Jaral del Progreso, Guanajuato. Nevertheless, several studies show that insects can adapt and incorporate new plants as food, although initially populations are low (Gassmann et al. 2006; Zhang et al. 2015; Messina et al. 2020). We suggest increasing the study area and sampling periodicity in strawberry-producing regions to study the association.

The genus *Argyrotaenia* Stephens includes around 116 species described worldwide (Powell 1983; Powell et al. 1995; Razowski 1996); of these, 115 species occupy habitats from Canada to Argentina (Obraztsov 1961; Brown 1999), the region of greatest species richness. Identification of *Argyrotaenia* is based mostly on external traits and genitalia. *Argyrotaeniamontezumae* shows an aedeagus slightly capitated, cornuti with thick tips, and a dilated coecum penis curved slightly downward (Fig. 2C, D) (Obraztsov 1961). Our finding concerning *A.montezumae* coincides with López et al. (2014) who report this species in blackberry crops (*Rubus* sp.) at elevations of 1350 m in Zamora, Michoacán. We also ratify that *A.montezumae* feeds on strawberry leaves (*F.×ananassa*), as indicated by Tejeda-Reyes et al. (2020). In our study area, *A.montezumae* is present in 83% of the studied orchards found at altitudes of up to 2337 m in blackberry, raspberry and strawberry fields. Therefore, it is undoubtable that this species is found in berry-producing areas of Mexico.

Finally, the genus *Platynota* includes 33 polyphagous species described and distributed on the American continent (Powell and Brown 2012). In our study, from a rolled blackberry leaf with a larva inside, an adult *Platynota* sp. emerged (Fig. 2E, F), thus corroborating that the genus *Rubus* is host to *Platynota*, as indicated by Gilligan and Epstein (2014), although it is necessary to extend the study area. Because of the small number of emerged specimens, it is difficult to assert which species we are dealing with. For this reason, we report it at the genus level. It is important to underline that several species of the genus have not been described despite its abundance in Central America (Brown 2013).

Our results extend the distribution of *A.cuneana* and *A.montezumae* to an elevation of 2337 m, without ruling out the possibility of finding them at lower or higher altitudes, wherever there are host plants since tortricids adapt better to temperate, subtropical and tropical climates (Meijerman and Ulenberg 2000), climates that coincide with the berry-growing regions of the country. Moreover, we can speculate that there may exist other tortricid species associated with berries, such as *Apotoforma* sp., which was found feeding on blackberry vegetative buds, flowers, and young fruits in Coatepec, Veracruz, Mexico (Ruiz 2019). However, in our study we did not find this species even though the elevation of this locality coincides with the lowest studied altitude. Morón and Terrón (1988) estimated that in Mexico there may exist 1500 species of tortricids, most have not been described.

## ﻿Conclusions

Three species of tortricids, *A.cuneana*, *A.montezumae* and *Platynota* sp., were identified associated with strawberries, raspberries, and blackberries in the producer regions of Michoacán and Guanajuato, Mexico, at altitudes from 1290 to 2337 m. The first two species were more abundant in the three crops, while *Platynota* sp. was observed only in blackberries. The three species belong to the subfamily Tortricinae, whose main characteristics are their behavior as leafrollers and their polyphagous feeding habit. In the three species of cultivated plants, both species were associated only with tender shoots and leaves. In our study, we did not quantify losses and damage from feeding. In later studies, measures for managing this group of insects should be designed, and the economic losses they cause to berry production in Mexico should be determined.
